# Delaying latency to hyperbaric oxygen‐induced CNS oxygen toxicity seizures by combinations of exogenous ketone supplements

**DOI:** 10.14814/phy2.13961

**Published:** 2019-01-03

**Authors:** Csilla Ari, Andrew P. Koutnik, Janine DeBlasi, Carol Landon, Christopher Q. Rogers, John Vallas, Sahil Bharwani, Michelle Puchowicz, Ilya Bederman, David M. Diamond, Mark S. Kindy, Jay B. Dean, Dominic P. D′Agostino

**Affiliations:** ^1^ Department of Psychology Hyperbaric Neuroscience Research Laboratory University of South Florida Tampa Florida; ^2^ Department of Molecular Pharmacology and Physiology Laboratory of Metabolic Medicine University of South Florida Tampa Florida; ^3^ Department of Molecular Pharmacology and Physiology Hyperbaric Biomedical Research Laboratory University of South Florida Tampa Florida; ^4^ Department of Pediatrics University of Tennessee Health Science Center Memphis Tennessee; ^5^ Department of Pediatrics Case Western Reserve University Cleveland Ohio; ^6^ Department of Nutrition Case Western Reserve University Cleveland Ohio; ^7^ Department of Pharmaceutical Sciences College of Pharmacy University of South Florida Tampa Florida; ^8^ James A. Haley VA Medical Center Tampa Florida; ^9^ Shriners Hospital for Children Tampa Florida; ^10^ Institute for Human and Machine Cognition Ocala Florida

**Keywords:** Acetoacetate, beta‐hydroxybutyrate, hyperbaric, ketogenic diet, ketone ester, oxygen toxicity seizures

## Abstract

Central nervous system oxygen toxicity (CNS‐OT) manifests as tonic‐clonic seizures and is a limitation of hyperbaric oxygen therapy (HBOT), as well as of recreational and technical diving associated with elevated partial pressure of oxygen. A previous study showed that ketone ester (1,3‐butanediol acetoacetate diester, KE) administration delayed latency to seizures (LS) in 3‐month‐old Sprague‐Dawley (SD) rats. This study explores the effect of exogenous ketone supplements in additional dosages and formulations on CNS‐OT seizures in 18 months old SD rats, an age group correlating to human middle age. Ketogenic agents were given orally 60 min prior to exposure to hyperbaric oxygen and included control (water), KE (10 g/kg), KE/2 (KE 5 g/kg + water 5 g/kg), KE + medium‐chain triglycerides (KE 5 g/kg + MCT 5 g/kg), and ketone salt (Na^+^/K^+^ *β* HB, KS) + MCT (KS 5 g/kg + MCT 5 g/kg). Rats were exposed to 100% oxygen at 5 atmospheres absolute (ATA). Upon seizure presentation (tonic‐clonic movements) experiments were immediately terminated and blood was tested for glucose and *D*‐beta‐hydroxybutyrate (*D*‐*β* HB) levels. While blood *D*‐*β* HB levels were significantly elevated post‐dive in all treatment groups, LS was significantly delayed only in KE (*P* = 0.0003), KE/2 (*P* = 0.023), and KE + MCT (*P* = 0.028) groups. In these groups, the severity of seizures appeared to be reduced, although these changes were significant only in KE‐treated animals (*P* = 0.015). Acetoacetate (AcAc) levels were also significantly elevated in KE‐treated animals. The LS in 18‐month‐old rats was delayed by 179% in KE, 219% in KE + MCT, and 55% in KE/2 groups, while only by 29% in KS + MCT. In conclusion, KE supplementation given alone and in combination with MCT elevated both *β* HB and AcAc, and delayed CNS‐OT seizures.

## Introduction

Hyperbaric oxygen (HBO_2_) is an FDA‐approved medical therapy for at least 14 conditions including air/gas embolism, decompression sickness, carbon monoxide poisoning, and diabetic wounds, among others. HBO_2_ involves breathing pressurized oxygen which results in increased dissolved oxygen in the blood plasma (Neuman and Thom 2008). This increased systemic partial pressure of oxygen (hyperoxia) helps deliver elevated levels of O_2_ to various tissues. However, hyperoxia can also generate excessive oxygen‐free radicals which can damage cellular components through oxidative stress, potentially leading to significant tissue injury. Consequently, prolonged HBO_2_‐induced hyperoxia has been shown to increase the risk, severity, and frequency of seizures (Clark and Thom 1997). This condition, which is a result of elevated tissue partial pressures of O_2_, is known as central nervous system oxygen toxicity (CNS‐OT), and occurs when breathing 100% O_2_ at barometric pressures (Pb) >2.4 atmospheres absolute (ATA), and poses a limitation on the use of HBO_2_ as a therapy (HBOT), and other applications of hyperbaric oxygen, such as recreational and technical divers using elevated O_2_ partial pressures.

HBO_2_ has additional applications within the field of research as a reliable, reproducible, and reversible stimulus for producing generalized tonic‐clonic seizures, proving useful for studying the physiological changes that occur during seizures, as well as for assessing the efficacy of various anti‐seizure methods in animal models. Rodent studies have shown that the development of hyperoxia‐induced seizures can be delayed by food deprivation. In a previous study, researchers demonstrated that fasting (24–36 h) could postpone the onset of seizures from HBO_2_ by up to 300% (Bitterman et al. 1997). Throughout episodes of fasting, or with strict adherence to a ketogenic diet (KD), the body limits glucose availability, which suppresses insulin signaling and mobilizes free fatty acids (FFA) for fuel from adipose tissue stores (Cahill 2006). Adipose‐derived FFAs are generally impermeable to the blood brain barrier (BBB), however; hepatic ketogenesis converts FFA into ketone bodies, *β*‐hydroxybutyrate (*β*HB), and acetoacetate (AcAc). Acetone is also produced in small amounts due to spontaneous decarboxylation of AcAc. Under normal conditions, the concentration of systemic *β*HB is very low (≤0.1 mmol/L) and accounts for <3% of total cerebral metabolism (Hawkins et al. 1971). Conversely, during extended periods of fasting or strict KD adherence, ketone bodies accumulate in the blood (up to ~5–6 mmol/L) and cross the BBB via monocarboxylic acid transporters (MCT1‐4), allowing for their utilization as fuel by the brain (Prins 2007). Under conditions of fasting it has been reported that >60% of brain energy metabolism is derived from ketone bodies *β*HB and AcAc (Cahill 2006).

Factors that increase oxidative stress may disrupt metabolic control in the brain, and ketone bodies may restore metabolic homeostasis through a broad array of biochemical, molecular, and cellular changes (Yao et al. 2011; Simeone et al. 2018). Multiple benefits have been linked to the metabolic adaptations associated with fasting‐induced ketosis, including decreased production of reactive oxygen species (ROS), improved mitochondrial function, reduction in inflammation, and expression of brain‐derived neurotrophic factor (BDNF) (Maalouf et al. 2009; Marosi et al. 2016). The clinical efficacy of the KD has been validated in several animal models of epilepsy. The KD increases the threshold for seizures induced by amygdala kindling and GABA antagonists (such as pentylenetetrazole), and delays the development of seizures in EL mice, SD rats, Frings audiogenic seizure‐susceptible, and flurothyl‐treated mice (Hori et al. 1997; Bough and Eagles 1999; Todorova et al. 2000; Mantis et al. 2004; Bough and Rho 2007; Rho and Sankar 2008). In rodents with kainic acid‐induced seizures, the KD reduces the risk of developing epilepsy, and the severity of the symptoms. These effects may be explained by the reduction in hippocampal excitability and decreased supragranular mossy fiber sprouting (Muller‐Schwarze et al. 1999; Noh et al. 2003; Xu et al. 2006). The improvement observed with ketone utilization is similar to that observed with therapeutic doses of anti‐epileptic drugs (AEDs) (Bitterman and Katz 1987; Tzuk‐Shina et al. 1991) and novel anticonvulsants that inhibit excitatory glutamatergic neurotransmission (Chavko et al. 1998).

Based upon these results and other studies, nutritional ketosis has been shown to be an effective treatment option for patients with drug‐resistant seizure disorders (Freeman and Kossoff 2010). The clinical use of therapeutic ketosis is well documented in both children and adults (Klein et al. 2010). Despite multiple studies demonstrating the efficacy of fasting and ketosis in seizure reduction, the molecular mechanisms have remained largely unknown. However, a recent study using absence seizure‐prone Wistar Albino Glaxo/Rijswijk (WAG/Rij) rats has proposed the involvement of the adenosinergic A1 receptor (AA1R) pathway (Kovács et al. 2017). An additional study suggests other stabilizing mediators could be involved, including polyunsaturated fatty acids, which modulate ion channels (Rogawski et al. 2016).

The anticonvulsant effects of the KD typically correlate with increased concentrations of ketone bodies in the blood, particularly AcAc and acetone (Bough and Rho 2007; Mcnally and Hartman 2012). However, when compared to prolonged fasting, the levels of blood ketones associated with the KD are often limited due to the difficulty of nearly complete carbohydrate restriction required (Cahill 2006). Ketone supplementation with esters of *β*HB or AcAc can induce a rapid and sustained therapeutic ketosis that mimics the effects of prolonged fasting or a rigid KD without dietary restriction (Desrochers et al. 1995; Brunengraber et al. 1997). Moreover, previous research has established that orally administered esters of *β*HB are tolerated and safe in rats (Clarke et al. 2012), as well as humans (Clarke et al. 2012). In 2013, D′Agostino et al. investigated whether a ketone diester [*R,S*‐1,3‐butanediol acetoacetate diester (BD‐AcAc_2,_ KE)] (Ciraolo et al. 1995; Desrochers et al. 1995; Puchowicz et al. 2000), could replicate fasting and the KD's ability to increase latency to seizure (LS). Using 3 months old SD rats, they showed that KE is effective in causing a rapid (<30 min) and sustained (> 4 h) elevation of AcAc (>3 mmol/L) and *β*HB (>3 mmol/L), and delayed LS by 574 ± 116% compared with control (water) due to the effect of AcAc and acetone, but not *β*HB alone (D'Agostino et al. 2007). During that study 5 ATA O_2_ pressure was tested. Therefore, to make our results comparable we used the same conditions for the present study. In a more recent study, the ability of KE to elevate blood *β*HB rapidly (<30 min) and for a sustained period (8 h) was further demonstrated in SD rats (Kesl et al. 2016).

Medium‐chain triglyceride (MCT) oil contains fatty acids 8–10 carbons in length. Upon ingestion, these medium‐chain fatty acids are readily converted to *β*HB causing a rapid elevation in blood ketones. It has been demonstrated that co‐ingestion of MCTs along with other ketogenic supplements prolonged ketone elevation (Kesl et al. 2016). Additionally, MCTs have been previously shown to have antiseizure effects (Augustin et al. 2018), therefore in this study we also tested ketogenic agents combined with MCTs.

The potential for ketone supplementation to circumvent the dietary restriction associated with the KD to achieve therapeutic ketosis has been previously discussed (Ari et al. 2015; D'Agostino 2016). This strategy to induce nutritional ketosis would be favored by those unwilling or unable to follow a strict KD. From an operational perspective, the use of ketone supplementation offers practical advantages due to the rapid onset and ability to titrate the dosage and formulation for individual response, pharmacokinetic profile and associated mechanistic properties (Bough and Eagles 1999; Kovács et al. 2017).

The specific aim of this study was to expand upon previous research establishing the link between therapeutic ketosis and the resulting antiseizure effects. We hypothesized that ketogenic strategies that elevate both *β*HB and AcAc would have the greatest potential delaying CNS‐OT. Thus, we explored the effect of different dosages and combinations of exogenous ketone supplements on CNS‐OT seizures in an older age group of male SD rats (18 months), which is used to model middle age in humans.

## Materials and Methods

### Animal procedures and treatment groups

All animal procedures were done in accordance with the University of South Florida Institutional Animal Care and Use Committee (IACUC) guidelines. All protocols were previously approved by the University of South Florida Institutional Animal Care and Use Committee (PHS Assurance No. A4100‐ 01; and fully accredited by AAALAC as Program No. 000434) and by the Director for Veterinary Affairs, Department of the Navy, Bureau of Medicine and Surgery.

Eighteen‐month‐old male SD rats (470–720 g, *n = *42, Harlan) were given normal rodent chow (Envigo; 19% protein; 75% carbohydrate; 6% fat) and water ad libitum and kept on a 12‐h light/dark cycle. The rats were assigned to one of five treatment groups: control (water, *n* = 9), 1,3‐ butanediol acetoacetate diester (BD‐AcAc_2_, KE, 10 g/kg, *n* = 9), KE/2 (KE, 5 g/kg + 5 g/kg water, *n* = 8) and KE + MCT (KE 5 g/kg + MCT 5 g/kg, *n* = 9). The ketone salt (KS) is formulated as a sodium/potassium‐ *β*HB salt (Na+/K+*β*HB) that is a 50% solution containing approximately 375 mg/g of pure D/L‐ *β*HB and 125 mg/g of Na+/K+. The Na+ and K+ are balanced to be in a 1:1 ratio. KS was mixed with equal parts MCT (KS 5 g/kg + MCT 5 g/kg, *n* = 7). The rats received one dose of their respective ketogenic agent by intragastric oral gavage 60 min prior to being exposed to hyperbaric conditions.

### Acquisition/analysis software

Raw video recordings were collected using DSI Ponemah software (version 4.90, P3 Ponemah Physiology Platform). GraphPad PRISM (version 3.03) was used for all statistical analyses. All values in this work were reported as means ± SEM of measurement. Analysis of data was performed using One‐way ANOVA and two‐tailed, unpaired t‐tests. Values were considered significant when *P* < 0.05. Prior to experiments it was determined that outliers (2SD) would be excluded if represented as mean data.

### Hyperbaric chamber

The hyperbaric system was composed of two main parts: Plexiglass chamber with a three‐liter capacity (Diamond Box, Buxco, Electronics Inc., model PLY3114) where the rat was kept during the experiment (kept at 100% O_2_), and a hyperbaric chamber pressurized with air (Reimers Systems, −7.8 ATA MWP) that held the Plexiglass chamber and operated as the pressure vessel. Pressuring only the Plexiglass chamber with 100% O2 averts the potential fire hazard of needing to pressurize the larger chamber with 100% O_2_. An air compressor (oil‐less rotary scroll compressor – model DK6086, Powerex) was connected to both chambers.

### HBO_2_ exposures (dive profile) and seizure detection

Thirty minutes after treatment, rats were placed in the hyperbaric chamber, where they were given 10 min to acclimate to the chamber, after which it was filled with pure oxygen, and the rats were allowed another 15 min to acclimate to the new atmosphere. The outer chamber was pressurized using air (capacity ~205 L). Next, both chambers were compressed to 5 atmospheres absolute (ATA) in parallel at a rate of approximately 1 ATA/min. The rat was visually monitored continuously via a live camera. Rats remained at 5 ATA until physical symptoms of CNS‐OT seizures were exhibited, as defined in the following section. LS was calculated from the moment at which the internal and the external chambers reached 5 ATA until the onset of convulsions (criteria listed below). After the onset of seizure, which was determined by a blinded experimenter, the Plexiglass chamber was flushed with air to quickly terminate the seizure, and both chambers decompressed to sea level at a rate of 1 ATA/min until gas within the chamber normalized to atmospheric pressure and composition.

Experiments were immediately terminated by the blinded experimenter if rats exhibited signs of pulmonary oxygen toxicity (observed as gasping and/or difficulty breathing) or exposure time at 5 ATA reached 120 mins. If either of those criteria were fulfilled, the inner chamber was flushed with air to reverse the effects of HBO_2_, and the chamber was decompressed at a rate of 1 ATA/min.

### Seizure definition and severity scores

Tonic‐clonic seizures were defined when rats exhibited two or more of the following symptoms: head bobbing up and down, blinking, elevation of one or two front paws, most often with opened toes or pushing the ground, that lasted for at least 10 s, or multiple and intense shakes or thrashing. A scoring system (ranging from 0 to 3) was used to record seizure severity (Table 1). A score of 0 indicated that no tonic‐clonic seizure was observed during the experiment. A score of 1 indicated that the animal showed mild symptoms, with seizures not lasting more than 10 sec and no signs of exhaustion shown post‐seizure. A score of 2 indicated that the animal displayed symptoms of an intense seizure for less than 10 sec, or subtle symptoms for longer than 10 sec. A score of 3 indicated that symptoms of a violent seizure were displayed, with intense, jerky movements for more than 10 sec, along with signs of exhaustion for an extended period post‐seizure.

**Table 1:** Definition of seizure severity categories used during the study

Severity score	Symptoms
0	No tonic‐clonic seizure was observed during the experiment
1	Mild and short (<10 sec) seizures. No postictal symptoms
2	Intense tonic‐clonic seizure for less than 10 sec or subtle symptoms for longer than 10 sec
3	Violent tonic‐clonic seizure for more than 10 sec. Postictal signs of exhaustion for an extended period

### Synthesis and formulation of ketogenic compounds

KE was synthesized as previously described by D'Agostino et al. (2013). KS is a novel agent that was mixed into a 50% solution supplying approximately 375 mg/g of pure *β*HB and 125 mg/g of Na^+^/K^+^ in a 1:1 ratio. Both KE and KS were developed and synthesized in collaboration with Savind (Seymour, IL). Human food grade MCT oil (~60% caprylic triglyceride/40% capric triglyceride) was purchased from Now Foods (Bloomingdale, IL). KS or KE were mixed with MCT in a 1:1 (by weight) ratio, generating the KS+MCT and KE+MCT combinations.

### Measurement and analysis of blood glucose and ketones

Blood was withdrawn from the rats via the saphenous vein. Blood concentrations of glucose and D‐*β*HB were determined utilizing a commercially available glucose and ketone monitoring system (Abbott Labs: Precision Xtra) immediately following removal from the hyperbaric chamber. Total blood *β*HB was estimated to be higher for KE and KS since this meter does not detect L‐*β*HB. In order to document the level of AcAc that was present in the animals when they were exposed to the hyperbaric oxygen 1 month later some of the animals (those remaining alive) received another treatment by oral gavage, and 60 min later whole blood samples (300 *μ*L) were collected into Eppendorf tubes in order to document AcAc levels that correlate to hyperbaric exposure time. Due to the limited number of aged animals, only control (water, *n* = 3), KE (*n* = 5), KE+MCT (*n* = 5) and KE/2 (*n* = 5) treatments were tested for AcAc and the animals were not matched with the treatment received during the previous experiment, so AcAc levels and LS was not correlated. Samples were processed for the detection and quantification of AcAc at Case Western Reserve University, Mouse Metabolic Phenotyping Center. Whole blood was collected, stabilized with cold 0.2 mol/L NaB^2^H_4_ to convert AcAc into [2‐^2^H] *β*HB, and then immediately frozen on dry ice. Samples were stored at −80°C until analyses. Briefly, internal standard of [2,4‐^13^C_2_] *β*HB was added to the treated blood samples (15 *μ*L) and *β*HB was extracted and converted to its trimethylsilyl (TMS) derivative by reacting lyophilized sample with 80 *μ*L of bis(trimethylsilyl) trifluoroacetamide + 10% trimethylchlorosilane (Regis, Morton Grove, IL) for 30 min at 75°C. di‐TMS derivative of *β*HB was analyzed by gas chromatography‐mass spectrometry (GC–MS) using an Agilent 5973 mass spectrometer, linked to a 6890 gas chromatograph equipped with an autosampler. Briefly, *β*HB (M/Z 233) was detected under the condition of electron ionization (EI) mode. M1 ion (M/Z 234) corresponding to [2‐^2^H] *β*HB represented AcAc amount present in the sample after appropriate background subtraction. M2 ion (M/Z 235) corresponded to an internal standard and was used to calculate AcAc and *β*HB sample concentrations.

## Results

### Latency to seizure and seizure severity

**Figure 1:**
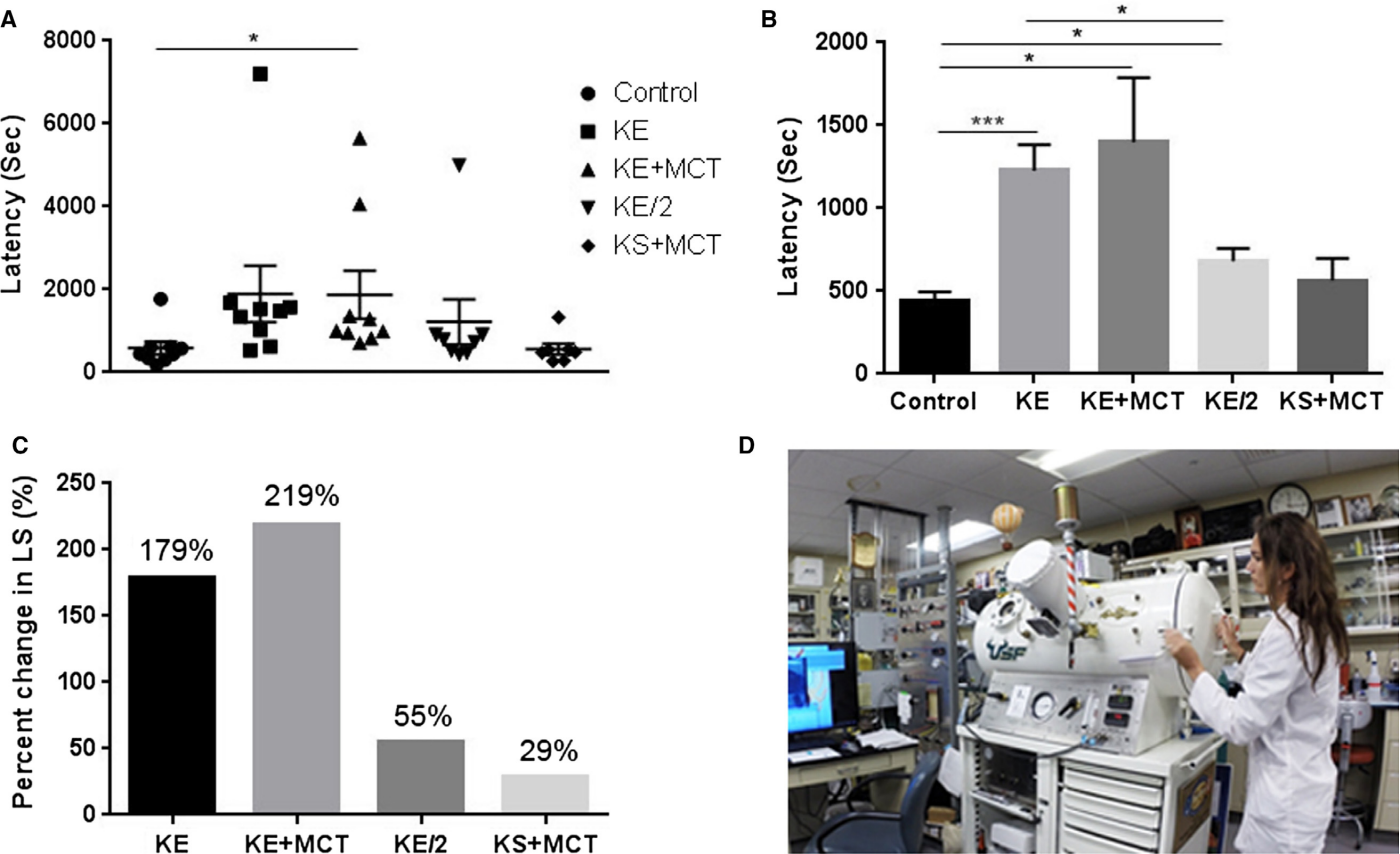
Delayed latency to seizure in response to ketone supplementation in Sprague‐Dawley rats. (A) A scatter plot (including outliers) demonstrating the latency to seizure (LS) for all specimens in the different treatment groups. KE + MCT group had significantly increased LS compared to control (*P* = 0.049). (B) The latency to seizure (LS) is presented after the exclusion of the outliers (2SD) from each treatment group. The LS was significantly delayed in the KE (*P* = 0.0003), KE+ MCT (*P* = 0.028) and KE/2 (*P* = 0.023) treatment groups when compared to control. LS in KE group was significantly higher than in KE/2 group (*P* = 0.011). (C) The percent change in latency to seizure (LS) when compared to control. (D) The blinded experimenter opening the hyperbaric chamber after determining seizure severity and performing gradual decompression.

**Figure 2:**
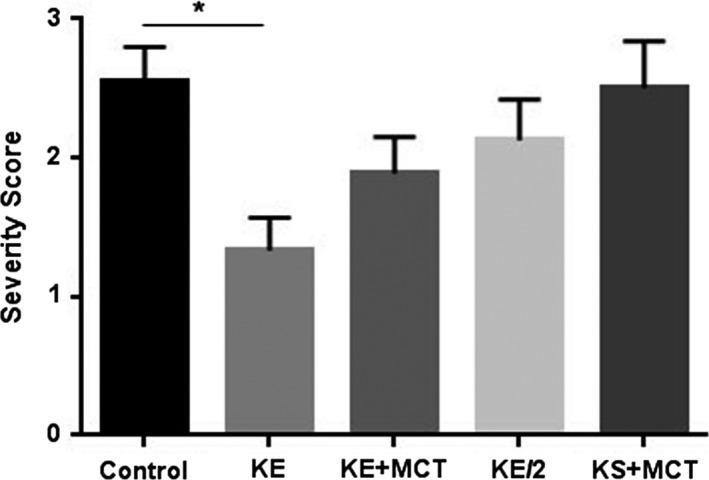
A bar graph displaying the severity of the seizure for all groups. The severity of seizures was significantly less in the KE group (*P* = 0.015), when compared to control.

Significantly delayed LS (in sec) from CNS‐OT was documented in KE+MCT group (*P* = 0.049; Fig. 1A), when all animals included in the experiment were considered. When outliers were removed (based on 2 standard deviation; Fig. 1B) LS was significantly delayed in the KE (*P* = 0.0003), KE/2 (*P* = 0.023), and KE+MCT (*P* = 0.028) groups. The KS+MCT treatment group showed a trend for delayed CNS‐OT, but was not significant when compared to control (water). It was determined that LS was also significantly different between KE and KE/2 groups (*P* = 0.011). In terms of percentage, the KE group experienced a delay to seizure of 179%, as compared to the control group, while the KE+MCT group showed a trend of greater anti‐seizure effect with LS = 219%, however, these groups were not statistically different (Fig. 1C). The KE/2 group delayed seizure by 55%, while the KS+MCT group showed a trend, but did not significantly influence LS. Moreover, the severity of seizures, determined by the blinded experimenter (Fig. 1D), was significantly less in KE (*P* = 0.015) and showed a trend towards less severity without significance in KE/2, and KE+MCT groups, when compared to control (Fig. 2). The LS in young and aged rats was not significantly different (*P* = 0.07).

### Blood *β*HB, AcAc, glucose, and body weight

**Figure 3:**
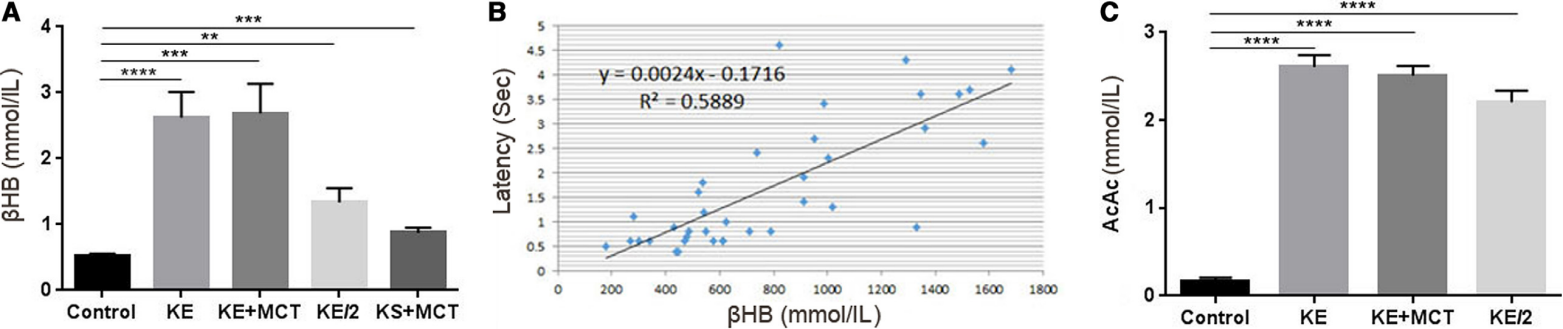
Changes in blood ketone levels in response to ketone supplementation. (A) All treatment groups showed a significant increase in blood D‐*β* HB concentrations. Compared to the control, the KE (*P* < 0.0001) and KE+MCT (*P* = 0.0002) groups had a higher level of D‐*β* HB by a factor of five, KE/2 group (*P* = 0.0012) by a factor of three, and the KS+MCT group (*P* = 0.0006) by a factor of approximately two. (B) There was a positive correlation between latency to seizure (LS) and blood D‐*β* HB concentration (*R* ^2^=0.5889). (C) The AcAc levels measured 60 min after treatment for the different treatment groups. The KE (*P* < 0.0001), KE+MCT (*P* < 0.0001) and KE/2 (*P* < 0.0001) groups had significantly elevated AcAc levels, compared to control.

**Figure 4:**
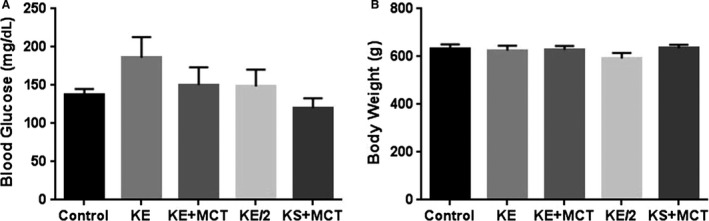
There was no significant difference in blood glucose levels (A) measured after removal from the chamber and in body weights (B) between the treatment groups and control.

All treatment groups experienced a significantly higher blood D‐*β*HB, compared to control (Fig. 3A**)**. Treatment with KE (*P* < 0.0001) and KE+MCT (*P* = 0.0002) resulted in higher *β*HB levels approximately five‐ to sixfold when compared to control. The KE/2 group (*P* = 0.0012) had higher *β*HB level then the control by a factor of approximately three, while it was about two times higher in the KS+MCT (*P* = 0.0006) group. There was a strong positive correlation between *β*HB levels and LS when all treatment groups were considered (*R* ^2^ = 0.588; Fig. 3B) and in the KE group alone (*R* ^2^ = 0.71). The elevation of *β*HB in KE‐containing treatment groups was also associated with an elevation of AcAc. AcAc was significantly higher in all treatment groups compared to control (*P* < 0.0001; Fig. 3C). In groups treated with KE, KE+MCT and KE/2 the AcAc levels were higher than control (water) by factors of 16, 15, and 14, respectively. Blood glucose and body weight were not significantly different between the treatment groups and control (Fig.**** 4).

## Discussion

In this study we examined the effect of different dosages and combinations of exogenous ketone supplements in the time‐course of CNS‐OT seizures, which remains a limitation of HBOT, as well as recreational and technical divers using enriched O_2_ mixes, including closed‐circuit rebreather diving technologies employed by the United States Navy Sea, Air, and Land (SEAL) operations. The LS was significantly delayed in the KE, KE/2 and KE+MCT groups and this correlated with significant elevations in both *β*HB and AcAc levels compared with control (water). The LS was not significantly delayed in the KS+MCT group (*β*HB precursors), which was associated with lower *β*HB levels and no elevation of AcAc. In addition, the severity of seizures was significantly less in the KE group and a trend towards lower severity could be observed in all other ketone supplement groups. In 3‐month‐old juvenile rats the KE delayed seizure by 574% compared with control (D'Agostino et al. 2013), while the LS in 18‐month‐old rats was delayed by 179% in the KE group, 219% in the KE + MCT group, and 55% in the KE/2 group. From these results, we demonstrated that rats in older age group, correlating to human middle age, showed delayed latency to CNS‐OT seizures. The LS was less pronounced than in younger rats, although still comparable to high‐dose, anti‐seizure drugs. Thus, we speculate that combining ketogenic supplements with AEDs, may produce more favorable effects on LS while mitigating adverse drug effects. The difference in LS observed in the two age groups may be due to age‐dependent alterations in O_2_ tolerance, changes in brain ketone metabolism, or changes in the neuropharmacological targets with aging. As the control groups in the two studies did not differ significantly in LS, this may provide support for the hypothesis that ketone metabolism and associated signaling change with age. One shortcoming of the study is that we have not yet characterized some of these ketone supplement formulations in younger rats. Further studies are needed to assess age‐related changes in ketone‐induced neuroprotection and to determine how chronic administration influences the neuroprotective antiseizure effects, as was done in other model systems (Kovács et al. 2017).

While all contributing mechanisms of KE‐induced neuroprotection against CNS‐OT still need to be elucidated, the findings from this study and other reports suggest a multifactorial contribution to the anti‐epileptic effect of ketone bodies (Simeone et al. 2018). The current study reinforces previous conclusions that seizure resistance is correlated with elevated ketone levels in the blood (Bough and Rho 2007; Mcnally and Hartman 2012). LS was significantly increased in KE compared to KE/2 group, suggesting a dose–response effect. Moreover, the trend of delayed seizure in KE+MCT group (219%), when compared to the KE group (179%), suggest that the addition of MCTs may provide an additive affect, however, more work would be necessary to confirm this. Further experiments are needed to examine the mechanisms associated with antioxidant neuroprotection (Maalouf et al. 2007; Kim et al. 2010; Milder et al. 2010) and enhanced metabolic efficiency (Veech 2004) that may also be contributing to these neuroprotective effects against the acute oxidative stress stimulus of HBO_2_.

The neuroprotective effect of fasting and the KD – specifically against seizures – has been well‐studied in animal models and humans, and was found to be positively correlated with levels of ketones (Bough and Rho 2007; Mcnally and Hartman 2012). However, both ketogenesis, and the seizure protection it confers, are reversed via consumption of carbohydrates or surplus protein. Therefore, exogenous ketone supplementation, such as those tested here, may be a more practical mitigation strategy for CNS‐OT, as well as a valuable option for epileptic patients who have issues adhering to the KD (Rho et al. 2002). Our findings suggest that the antiseizure effect of therapeutic ketosis induced with supplementation is observed even in older rats eating a standard carbohydrate‐based diet.

### Effects of the ketone intervention groups

It has been proposed that *β*HB alone, the primary ketone in circulation, does not impact CNS‐OT in a significant manner if elevated independently from AcAc. However, *β*HB elevated with both AcAc and acetone has been shown to delay seizures in CNS‐OT (D'Agostino et al. 2013) and in other animal models of seizures (Likhodii et al. 2008; Rho and Sankar 2008). All treatment groups that experienced a delay of CNS‐OT received KE, which is a precursor to *β*HB, AcAc and acetone. There was a strong positive correlation between blood *β*HB levels and LS in the KE group (*R* ^2^ = 0.711) and when all KE‐associated treatment groups were considered (*R* ^2^ = 0.588), presumably due to KE‐induced increases in AcAc and acetone (D'Agostino et al. 2013). While D‐*β*HB was elevated within the serum of treated animals, it is important to note that the KE and KS used in this study are composed of racemic mixtures resulting in elevations of both D‐*β*HB and L‐*β*HB. Thus, the reported value of measured *β*HB only reflects that of D‐*β*HB, not of total *β*HB. Similarly, KE, KE/2, and KE+MCT groups had significantly higher AcAc levels compared to the control. Acetone was previously reported to play a potential antiseizure role against CNS‐OT with KE administration. It can be estimated that approximately 20% of AcAc spontaneously decarboxylates to acetone (D'Agostino et al. 2013), but since there is enzymatic interconversion between *β*HB and AcAc it becomes difficult to attribute the anti‐seizures effects to AcAc or acetone alone. Indeed, acetone alone is comparable or better than some broad‐spectrum anticonvulsants (Likhodii et al. 2008), suggesting it may have contributed independently to the observed antiseizure effects.

### Ketone‐induced neuroprotection

There is increasing experimental evidence that ketone‐induced anti‐seizure properties function through a multiplicity of mechanisms associated with suppression of neuronal hyperexcitability, inflammation, and redox stress. These mechanisms include but are not limited to: (Ari et al. 2015) activation of inhibitory adenosine and ATP‐sensitive potassium channels; (Augustin et al. 2018) enhancement of mitochondrial function and reduction in oxidative stress; (Bitterman and Katz 1987) attenuation of neuronal excitability by modulating vesicular glutamate transporters or VGLUTs (notably, AcAc), and (Bitterman et al. 1997) enhancement of central *γ*‐aminobutyric acid (GABA) synthesis. Other novel actions more recently reported include inhibition of NLRP3 inflammasome assembly and decreasing the activity of histone deacetylases (HDACs). This study supports the in vivo antiseizure effects of *β*HB, AcAc, and ACE, as reported previously (Simeone et al. 2018). However, the question of whether one specific ketone or a combination of these ketone bodies affords even greater efficacy has not yet been answered.

### Ketone ester‐induced metabolic therapy

Another proposed mechanism by which ketones delay CNS‐OT involves enhancing mitochondrial efficiency (Veech 2004). It has been well established that ketones can replace glucose as the primary fuel (>50%) for brain energy metabolism during periods of limited glucose availability resulting from starvation or carbohydrate restriction (Cahill 2006). Previous studies in rats show that starvation delays the onset of CNS‐OT (Bitterman et al. 1997), presumably via a fundamental shift in brain energy metabolism away from glucose utilization. Starvation (24–36 h) also delays the latency to seizure from HBO_2_ by up to 300%, which is equally – or more effective than – high doses of clinically approved anti‐epileptic drugs (AEDs) (Bitterman and Katz 1987; Tzuk‐Shina et al. 1991) or experimental anticonvulsants that block excitatory glutamatergic neurotransmission (Chavko et al. 1998). Based upon the hypothesis that CNS‐OT is the result of metabolic dysfunction, secondary to hyper‐excitability and oxidative stress from hyperoxia, therapeutic ketosis should be considered as a metabolic‐based therapy. This study identifies several exogenous ketone supplement formulas that show therapeutic efficacy against CNS‐OT and would be likely candidates for translational studies in human subjects.

## Conclusions

Previous experiments have established the relationship between *β*HB, AcAc, and seizure resistance in younger rats. This study extends those findings and demonstrates the utility of additional combinations of ketone supplementation as a practical strategy for mitigating CNS‐OT in a rodent age group that rather models middle‐age humans. These results indicate that potentially new combinations of ketone supplements, specifically KE‐based formulas, that elevate both *β*HB and AcAc, can be used to effectively delay seizures. Further advantage of these ketone supplement formulas may be better palatability, safety, and effectiveness; therefore, we urge further investigation of these compounds as potential therapeutics for resilience against CNS‐OT.

## Conflict of Interest

International Patent # PCT/US2014/031237, University of South Florida, D. P. D'Agostino, S. Kesl, P. Arnold, “Compositions and Methods for Producing Elevated and Sustained Ketosis”. Non‐provisional patent: C. Ari, D. P. D’Agostino, J. B. Dean, Technology Title: “Delaying latency to seizure by combinations of ketone supplements”. USF Ref. No: 16B138PR. D. P. D'Agostino and C. Ari are co‐owners of the company Ketone Technologies LLC. These interests have been reviewed and managed by the University in accordance with its Institutional and Individual Conflict of Interest policies. All authors declare that there are no additional conflicts of interest.
